# Preventative Measures for Lower Extremity Skin Conditions in Paralympic and Adaptive Sports: An Epidemiological Overview

**DOI:** 10.26502/aimr.0229

**Published:** 2025-12-18

**Authors:** Vera Wang, Andre Aabedi, Devendra K. Agrawal

**Affiliations:** 1 Department of Translational Research, College of Osteopathic Medicine of the Pacific, Western University of Health Sciences, Pomona, California 91766 USA

**Keywords:** Adaptive athletes, Dermatology, Disabilities, Infection prevention, Paralympic athletes, Prosthetics, Skin infections, Tele-dermatology

## Abstract

Adaptive and Paralympic athletes face unique dermatologic challenges related to impaired sensation, prosthetic use, wheelchair friction, and comorbid conditions. Lower extremity skin infections are particularly concerning due to their impact on performance, participation, and overall health. To review the epidemiology, risk factors, clinical features, and evidence-based prevention strategies for lower extremity skin infections in adaptive and Paralympic athletes, and to identify current research gaps and future directions. A narrative epidemiological review was conducted using data from Paralympic Games surveillance systems, sports medicine registries, and dermatologic literature on skin infections in athletes with disabilities. Relevant studies addressing prevalence, pathophysiology, and preventive interventions were synthesized. Skin and soft tissue infections occur at a higher rate in adaptive sports athletes compared to the general population, with the highest rates in individuals with spinal cord injury and prosthetic use. Key risk factors include compromised skin barrier integrity, impaired circulation, hygiene challenges, and environmental exposure. Prevention requires a multifaceted approach emphasizing daily skin inspection, hygiene optimization, prosthetic fit adjustments, and facility disinfection. Multidisciplinary education of athletes, coaches, and clinicians is critical for early recognition and intervention. Despite the high burden, dermatologic outcomes remain underreported, and few studies evaluate targeted preventive measures. Lower extremity skin infections are a prevalent and preventable cause of morbidity in adaptive and Paralympic athletes. Tailored dermatologic care, standardized surveillance, and technological innovations—such as antimicrobial prosthetic liners and AI-assisted tele-dermatology—offer promising avenues to reduce infection burden and promote inclusion in sport. Future research should prioritize longitudinal, multicenter studies to inform evidence-based prevention and management strategies.

## Introduction

Exercise is beneficial not only for physical health, but also for psychological well-being and social integration. Individuals with physical disabilities face barriers to participation resulting in substantially lower levels of physical activity than those of the general population [1,2]. Adaptive sports, which offer modifications for physical, sensory, or intellectual disabilities ranging from noncompetitive recreational activities to high-performance events like the Paralympics, have been a catalyst for inclusion and accessibility [3]. These programs are structured to promote physical fitness, social integration, and psychosocial well-being, and are recognized as agents of social change and empowerment for people living with disabilities [4].

Skin integrity is critically important for both performance and health in these athletes. Individuals with spinal cord injuries or who use wheelchairs and prostheses are especially at risk for adverse outcomes from repetitive friction, pressure, and impaired sensation, leading to skin breakdown. These can progress to infections if untreated, with complications ranging from inability to participate in training or competitions to more serious conditions such as osteomyelitis or autonomic dysreflexia [5]. This review aims to summarize epidemiologic trends, identify risk factors, and discuss prevention strategies for lower extremity skin infections in adaptive sports athletes.

### Epidemiology of Skin Infections in Adaptive and Paralympic Athletes

#### Prevalence and Incidence

Skin infections are common in athletes, with an even higher prevalence in adaptive athletes [6]. Skin infections account for approximately 21% of health conditions and injuries in collegiate sports and 8.5% in high school sports in the general population, with the highest rates in football and wrestling. Systematic review and meta-analysis suggest that illness incidences, including skin infections, are highest in Paralympic athletes compared to Olympic and Youth Olympic athletes [7]. During the London 2012 Paralympic Games, skin and subcutaneous illnesses accounted for 11.8% of all reported illnesses, with the highest rates seen in athletes with spinal cord injury (up to 18% of illnesses in this group [8]. At the Rio 2016 Paralympic Games, the incidence rate for skin and subcutaneous system illnesses was 1.8 per 1000 athlete days, and at the Tokyo 2020 Paralympic Games, dermatological illnesses had the highest incidence among all organ systems (1.1 per 1000 athlete days) [9,10]. Overall, epidemiologic studies consistently demonstrate a high incidence and burden of skin and soft tissue infections in this population, with infection rates and time-loss days exceeding those seen in able-bodied athletes.

#### Types of Skin Infections

The main types of skin infections among athletes—including both adaptive/Paralympic and able-bodied athletes—are bacterial, fungal, and viral infections. Bacterial infections are the most common, accounting for 60.6% of skin infections in US high school athletes. These are predominantly caused by Staphylococcus aureus (including Methicillin-Resistant Staphylococcus Aureus) and Streptococcus species, presenting as impetigo, folliculitis, cellulitis, and abscesses [11]. Fungal infections, representing 28.4% of skin infections in high school athletes, are primarily dermatophyte infections such as tinea corporis and tinea pedis caused by Trichophyton species [12]. Viral infections are less common and include herpes simplex virus, molluscum contagiosum, and human papillomavirus [13].

Potential complications of these infections include abscess formation, cellulitis, systemic spread (e.g., sepsis), and secondary bacterial infection of viral or fungal lesions. In adaptive and Paralympic athletes, especially those with spinal cord injury or impaired sensation, complications can be more severe, including deep tissue infection, osteomyelitis, and autonomic dysreflexia triggered by skin infection or breakdown [14]. MRSA infections are notable for their resistance to standard antibiotics and risk of outbreaks in team settings [15].

#### Pathophysiology and Risk Factors

Both intrinsic and extrinsic risk factors contribute to higher rates of skin infections in adaptive sports athletes. Intrinsic complications include impaired sensation and circulation, skin barrier compromise, and immunocompromised state, and extrinsic risk factors include the use of prosthetic devices and liners and hygiene challenges.

Impaired sensation, as seen in spinal cord injury, leads to unrecognized skin trauma and delayed detection of infection or ulceration. Loss of neuronal control alters cellular and molecular skin homeostasis, increasing plasma leakage, transforming growth factor-β activity, and inappropriate wound healing, which predisposes to pressure ulcers and secondary infection [16]. Poor circulation, common in diabetes and peripheral vascular disease, impairs tissue perfusion and immune cell delivery, increasing susceptibility to complicated skin and soft-tissue infections and reducing healing capacity [17].

Skin barrier compromise from repetitive pressure, shear, friction, or microtrauma is frequent in athletes using prosthetics, wheelchairs, or engaging in contact sports. This creates portals of entry for pathogens and increases risk for bacterial, fungal, and viral infections. Moist environments, maceration, and frequent skin trauma further disrupt the barrier function [18].

Immune compromise, whether due to underlying conditions or medication effects, reduces host defense against pathogens and increases infection risk and severity. The American College of Sports Medicine highlights compromised host immune status as a key risk factor for skin infections in athletes [19].

Hygiene challenges, such as limited access to bathing facilities or need for assistance with personal care, are significant extrinsic risk factors. Poor personal hygiene, infrequent bathing, and inadequate handwashing are associated with higher rates of skin infections, especially in athletes with disabilities who may require help with these tasks [20].

Overall, athletes with impaired sensation, poor circulation, compromised skin barrier, hygiene challenges, or immune dysfunction are at increased risk for skin infections and complications, with the risk amplified in those with spinal cord injury, diabetes, or who use assistive devices.

#### Clinical Presentation and Diagnosis

The typical clinical presentation of skin infections in the lower extremities of athletes using prosthetic devices includes erythema, warmth, swelling, tenderness or pain, purulent drainage, ulceration, and prosthetic discomfort. In this population, infection may also manifest as increased prosthetic intolerance, foul odor, delayed wound healing, or new onset of drainage at the skin-prosthesis interface. Systemic signs such as fever or chills are less common but indicate more severe infection [21,22]. The most effective evidence-based criteria for differentiating infection from non-infectious skin injuries in athletes who use prosthetic devices are the presence of purulent exudate or at least two signs of inflammation (erythema, pain/tenderness, induration, heat, or edema), as recommended by the Infectious Diseases Society of America [23]. However, in chronic wounds and in populations with impaired sensation (such as many adaptive athletes), classic signs alone have limited sensitivity and specificity, and clinical context is essential [24].

Key diagnostic considerations in distinguishing infection from pressure-related or friction-related skin injury involve careful clinical assessment. Pressure or friction injuries often present as non-infected ulcers, blisters, or calluses without significant erythema, warmth, or purulent drainage. In contrast, infection is suggested by the presence of purulent exudate, spreading erythema, increased warmth, edema, and pain [22]. In athletes with impaired sensation, pain may be absent, so increased drainage, malodor, and delayed healing are more reliable indicators. Ulcers with exposed bone or deep tissue should raise suspicion for underlying osteomyelitis [21].

Swab cultures are of limited value for superficial wounds, as they often reflect colonization rather than true infection. The Infectious Diseases Society of America and the American Society for Microbiology recommend that quantitative swab cultures using the Levine technique or deep tissue biopsy cultures are preferred for diagnosing infection in chronic wounds or ulcers, especially when infection is suspected but not clinically obvious. Surface swabs should be avoided for routine diagnosis [22]. Wound assessment tools (e.g., standardized scoring systems for infection severity) and imaging can aid in diagnosis and management [21,25].

#### Prevention and Management Strategies

Recommended prevention and management strategies for skin infections and complications in adaptive sports athletes include a multifaceted approach integrating hygiene, education, equipment modification, environmental controls, medical management, and multidisciplinary collaboration.

Hygiene is key to preventing skin infections, however individuals with disabilities may face additional barriers to maintenance. Daily skin checks are essential for early detection of erythema, warmth, drainage, ulceration, or prosthetic discomfort, especially in areas of impaired sensation or circulation. Cleansing with mild soap and water, followed by thorough drying particularly in skin folds and under prosthetic liners reduces moisture and microbial colonization. Regular moisturizing with non-comedogenic, fragrance-free emollients helps maintain skin barrier integrity and prevent fissures. Athletes should shower after training, trim nails, and practice meticulous hand hygiene [19].

Proper prosthetic fitting minimizes friction and pressure injuries, reducing risk of skin breakdown and infection. Use of antimicrobial liners and breathable fabrics under prosthetics and wheelchairs is recommended to decrease heat and moisture accumulation [26]. Routine cleaning and disinfection of prosthetic devices and wheelchairs, as well as immediate laundering of uniforms and towels, are necessary to prevent fomite transmission [19].

Infection control protocols in training facilities should include regular cleaning of shared surfaces and equipment with hospital-grade disinfectants [19,27]. Screening and isolation procedures are required during outbreaks, with exclusion from play until lesions are noninfectious and adequately treated. Routine surveillance and documentation of skin lesions facilitate early identification and containment [19].

Early treatment of minor lesions with topical or systemic agents, as appropriate, is recommended to prevent progression and transmission [28]. Barrier creams or antimicrobial wipes may be used for high-risk areas, especially under prosthetic liners [19]. Prophylactic medications can be considered for athletes with frequent outbreaks, following clinical guidelines. Return-to-play decisions should be based on evidence-based criteria, and infectious disease consultation is warranted for severe or refractory cases [18].

Education for both athletes and caregivers should focus on recognizing early signs of infection and the importance of prompt reporting and intervention [27]. Training in skin care, hygiene, and equipment maintenance is critical for both athletes and caregivers, with emphasis on daily inspection and cleaning routines [26].

Optimal prevention and management require collaboration among dermatologists, physiatrists, prosthetists, trainers, and athletes, ensuring comprehensive care and individualized risk assessment. This team approach facilitates education, surveillance, equipment modification, and prompt intervention. These strategies, grounded in consensus statements from the American College of Sports Medicine and supported by clinical studies, are essential for reducing the incidence and severity of skin infections and complications in athletes using prosthetic devices [19].

## Discussion

Although adaptive sports athletes are at an increased risk for skin infections, there is a lack of research focusing specifically on this population. Current gaps in the literature include a lack of large-scale epidemiologic data, few interventional or longitudinal studies on infection prevention, and limited inclusion of dermatologic outcomes in sports medicine research.

There is a scarcity of robust, multicenter, longitudinal epidemiologic studies specifically focused on skin infections and dermatologic outcomes in adaptive and Paralympic athletes. Most available data are derived from single events (e.g., Paralympic Games) or limited national cohorts, and often lack granularity regarding impairment type, sport modality, and environmental exposures. Systematic reviews confirm that the certainty of evidence for illness and injury rates in Paralympic athletes is low compared to Olympic cohorts, and dermatologic outcomes are infrequently reported as primary endpoints [7,29].

Additionally, the literature is dominated by cross-sectional or surveillance studies, with very few prospective interventional trials evaluating the effectiveness of specific prevention strategies in this population. There is limited evidence on the impact of targeted education, environmental controls, or multidisciplinary approaches on reducing skin infection rates or improving dermatologic health in adaptive and Paralympic athletes. Most current research is in able-bodied athletes, missing the additional barriers adaptive athletes face [29].

Most sports medicine research prioritizes musculoskeletal injuries and general illness, with dermatologic outcomes rarely included as primary or secondary endpoints. Surveillance systems and consensus statements, including those from the American College of Sports Medicine, often group skin infections with other illnesses and do not provide detailed dermatologic characterization or outcome measures [8,19]. Further studies focusing on dermatologic diagnoses would help better elucidate the prevalence and outcomes of these conditions.

Recommended future directions for standardized surveillance of skin infections in adaptive sports include the development of comprehensive, routine monitoring protocols tailored to the unique risk profiles of adaptive and Paralympic athletes, incorporating detailed skin assessments at preparticipation and ongoing intervals to identify early lesions and infection outbreaks, as emphasized by the American College of Sports Medicine consensus on illness prevention [19]. Surveillance systems should integrate standardized documentation of lesion type, location, and severity, with particular attention to athletes using prosthetic devices or with impaired sensation.

Prosthetic innovation offers opportunities to reduce skin infection risk by advancing antimicrobial liners, breathable and moisture-wicking materials, and pressure-distributing designs that minimize friction and skin barrier compromise. These innovations can be coupled with embedded sensors to monitor temperature, moisture, and pressure in real time, enabling early detection of skin breakdown before clinical infection develops.

Tele-dermatology, enhanced by artificial intelligence (AI), represents a promising avenue for scalable, remote skin surveillance in adaptive athletes. AI-driven image analysis can improve diagnostic accuracy, triage, and monitoring of skin lesions via smartphone or specialized imaging devices, facilitating timely intervention without requiring in-person visits [30]. Integration of AI with tele-dermatology platforms can support clinicians by providing decision support, confidence metrics, and longitudinal tracking of wound progression.

AI-based monitoring tools, including machine learning algorithms for wound detection and classification through imaging, can automate and standardize surveillance, improving early infection detection and personalized management [31]. These tools can be embedded in mobile applications or prosthetic device interfaces, enabling continuous monitoring and patient engagement. However, challenges remain regarding validation, data privacy, regulatory frameworks, and equitable access, necessitating multidisciplinary collaboration to optimize implementation [32].

In summary, future surveillance in adaptive sports should combine standardized clinical protocols with prosthetic technological advances and AI-augmented tele-dermatology to enhance early detection, prevention, and management of skin infections in this high-risk population

## Conclusion

Lower extremity skin infections represent a significant yet underrecognized health concern among adaptive and Paralympic athletes. This population faces unique dermatologic vulnerabilities arising from impaired sensation, altered biomechanics, prosthetic and wheelchair use, moisture retention, and comorbidities such as diabetes and vascular disease. Epidemiologic studies consistently demonstrate that skin and soft tissue infections occur at higher rates and with greater morbidity in adaptive athletes compared to able-bodied peers, leading to substantial training interruptions and potential long-term complications.

Evidence-based prevention centers on early detection, meticulous hygiene, equipment optimization, and multidisciplinary education. Routine skin checks, proper prosthetic fit and maintenance, environmental disinfection, and athlete education remain the cornerstones of infection control. Tailoring these strategies to individual impairment types and functional needs is essential to achieving equitable dermatologic care.

Despite advances in adaptive sports medicine, critical gaps persist in dermatologic surveillance and intervention research. There is a pressing need for large-scale, longitudinal studies to better characterize incidence, risk stratification, and response to preventive measures in this population. Future work should integrate technological innovations—such as antimicrobial prosthetic materials, wearable sensors, and AI-assisted teledermatology—to enable early detection and personalized prevention at scale.

Ultimately, promoting dermatologic health in adaptive and Paralympic athletes is not only a medical priority but also an issue of inclusion, performance, and dignity. Ensuring access to tailored skin care and infection prevention strategies empowers athletes to participate safely, sustain performance, and fully realize the physical, psychological, and social benefits of sport.

## Figures and Tables

**Figure 1:**
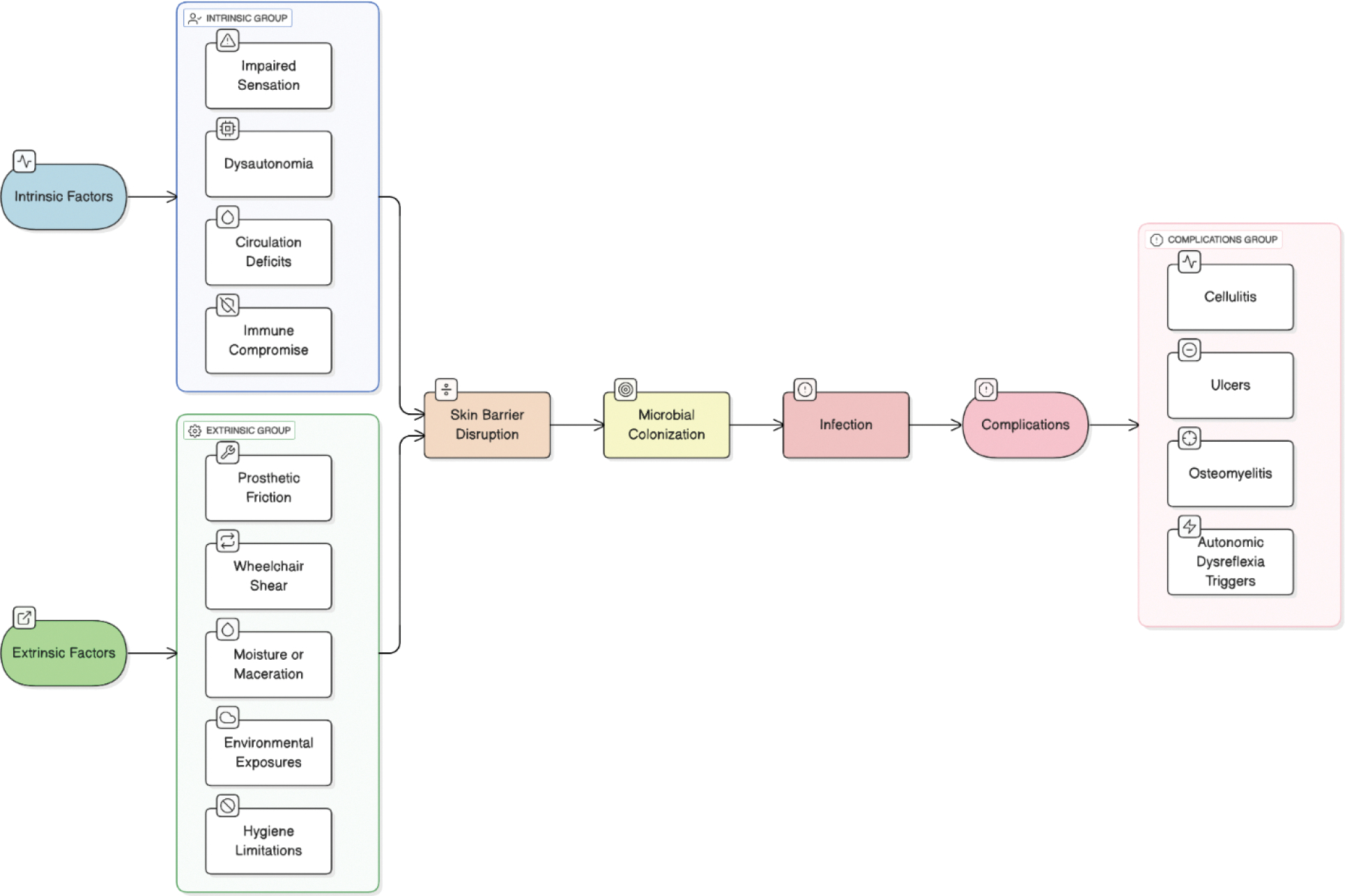
Risk pathways for skin infections in adaptive and paralympic athletes.

## Key points:

- Adaptive and Paralympic athletes experience disproportionately high rates of lower extremity skin infections compared with able-bodied athletes, especially those with spinal cord injury or prosthetic use.
- Impaired sensation, reduced circulation, skin barrier compromise, prosthetic friction, wheelchair-related shear forces, and hygiene challenges significantly elevate infection risk.
- Bacterial infections, particularly with Staphylococcus aureus (including MRSA) and Streptococcus species, are most prevalent, followed by dermatophyte fungal infections and less frequent viral infections.
- Traditional signs of infection may be muted in athletes with impaired sensation, making indicators such as drainage, malodor, prosthetic intolerance, and delayed healing especially important for diagnosis.
- Proper prosthetic fit, routine liner cleaning, moisture control, breathable fabrics, and use of antimicrobial prosthetic materials help reduce friction, maceration, and microbial growth.
- Consistent bathing, thorough drying, emollient use, facility disinfection, and laundering of equipment/clothing are essential to decrease fomite transmission and maintain barrier health.
- Effective prevention requires coordinated efforts among dermatologists, physiatrists, prosthetists, trainers, caregivers, and athletes for education, surveillance, and timely intervention.
- Dermatologic outcomes in adaptive sports are underreported, with limited longitudinal or interventional studies evaluating targeted prevention strategies for this population.
- Antimicrobial prosthetic liners, wearable sensor technologies, AI-enhanced tele-dermatology, and standardized surveillance systems offer promising avenues for earlier detection and personalized infection prevention.
